# Disseminated Salmonella Infection in an Immunocompromised Patient

**DOI:** 10.7759/cureus.26922

**Published:** 2022-07-16

**Authors:** Coulter Small, Adam Bria, Nayrobi M Pena-Cotui, Norman Beatty, Alaina S Ritter

**Affiliations:** 1 Division of Infectious Diseases and Global Medicine, University of Florida College of Medicine, Gainesville, USA

**Keywords:** gastroenteritis, immunocompromise, necrotizing fasciitis, septic arthritis, salmonella infection

## Abstract

*Salmonella *infection is a major public health concern worldwide. While non-typhoidal *Salmonella *serovars typically present with gastroenteritis, a disseminated infection may occur in high-risk individuals. After the initial invasion of the gastrointestinal mucosa, *Salmonella* spp. are capable of hematogenous dissemination throughout the body, leading to significant morbidity and mortality. We present a case of an immunocompromised patient with lower extremity abscesses, septic arthritis, and necrotizing fasciitis to highlight an uncommon presentation of disseminated *Salmonella* infection.

## Introduction

*Salmonella *spp. are gram-negative intracellular pathogens that are a leading cause of morbidity and mortality worldwide [1]. Following ingestion, *Salmonella *spp. invade the intestinal epithelium and typically cause gastrointestinal symptoms [2]. This organism also has the potential to disseminate and cause severe disease, especially in immunocompromised individuals [3,4]. In addition, there is growing concern regarding the development of antimicrobial resistance among *Salmonella *spp. [5-7]. In this case, we describe a unique presentation of disseminated *Salmonella *infection that illustrates the potential risk posed to immunocompromised patients.

## Case presentation

A 72-year-old man initially presented to his local emergency department with complaints of progressively worsening left hip pain. The pain developed after working in his garden and was associated with concomitant erythema and edema of the left thigh, as well as difficulty with ambulation. He denied a history of trauma, nausea, vomiting, diarrhea, changes in bowel function, fever, intravenous drug use, or recent travel.

His past medical history was notable for rheumatoid arthritis, Sjögren’s syndrome, and a total left hip arthroplasty performed 30 years prior. His immunosuppressive regimen consisted of methotrexate 22.5 mg orally once weekly, tocilizumab 162 mg subcutaneous injection once weekly, and prednisone 3 mg orally daily. He denied a history of animal contact or consumption of undercooked animal products.

**Figure 1:**
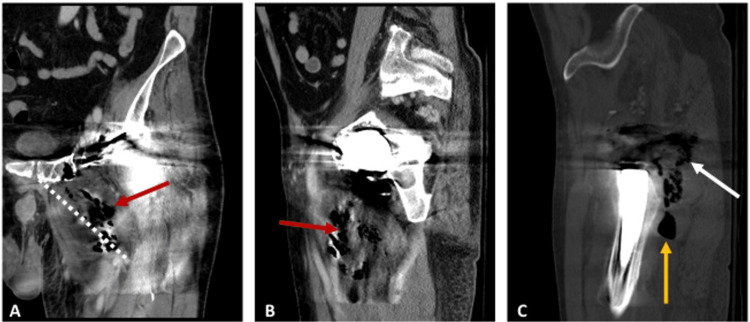
Original left hip computed tomography scan from the outside hospital (A) Coronal view of the largest abscess collection (red arrow), measuring 8.3 x 3.7 x 6.7 cm and tracking from the symphysis pubis to the middle thigh (dashed-white line). (B) The aforementioned abscess is viewed from a sagittal image. (C) Severe cellulitis and diffuse myofascitis (white arrow) and additional abscess (gold arrow) in the buttock and posterior thigh.

On presentation to his local emergency department, plain radiographic films of the left hip demonstrated no acute abnormalities. He was discharged five days later, with coordinated follow-up with his rheumatologist, because he was afebrile and nontoxic-appearing. At his rheumatology appointment, he was referred back to the emergency department due to ongoing symptoms and leukocytosis. A CT scan of the left lower extremity performed in the emergency department showed multifocal intramuscular peripheral abscess collections containing gas and fluid in the left posterior gluteal region, extending to the left greater trochanteric bursa region and the left abductor musculature. The largest abscess collection measured 8.3 x 3.7 x 6.7 cm with severe cellulitis and diffuse hamstring myofascitis tracking from the symphysis pubis to the middle thigh (Figure 1).

Additional CT findings included extensive particle granulomatous disease, osteolysis, and hardware loosening surrounding the left acetabular cup. He was started empirically on vancomycin and piperacillin/tazobactam, and the decision was made to transfer him to our academic institution for further management.

Upon presentation to our institution, the patient was afebrile and hemodynamically stable. He had a temperature of 36.7°C, blood pressure of 125/79, and a SpO2 of 94%. Laboratory findings are displayed in Table 1.

**Table 1:** Initial blood test results

Marker	Reference Value	Patient’s Value
White blood cell count	4.0 – 10 x 10^3 ^cells/mm^3^	19.0 x 10^3 ^cells/mm^3^
Neutrophils	40.0 – 80.0%	91.6%
Lymphocytes	20.0 – 45.0%	4.6%
Monocytes	2.0 – 10.0%	2.8%
Eosinophils	0.0 – 8.0%	0.7%
Basophils	0.0 – 2.0%	0.3%
Platelet count	150 – 450 x 10^3 ^cells/mm^3^	162 x 10^3 ^cells/mm^3^
Hemoglobin	13.0 – 16.5 g/dL	13.4 g/dL
Hematocrit	39.0 – 49.0%	40.7%
Albumin	3.5 – 5.2 g/dL	2.5 g/dL
Creatinine	0.5 – 1.2 mg/dL	0.93 mg/dL
Blood urea nitrogen (BUN)	6 – 21 mg/dL	32 mg/dL
Aspartate aminotransferase (AST)	0 – 37 IU/L	39 IU/L
Alanine aminotransferase (ALT)	0 – 50 IU/L	77 IU/L
Total Bilirubin	0.0 – 1.0 mg/dL	1.2 mg/dL
Alkaline Phosphatase	40- 150 IU/L	143 IU/L

**Figure 2:**
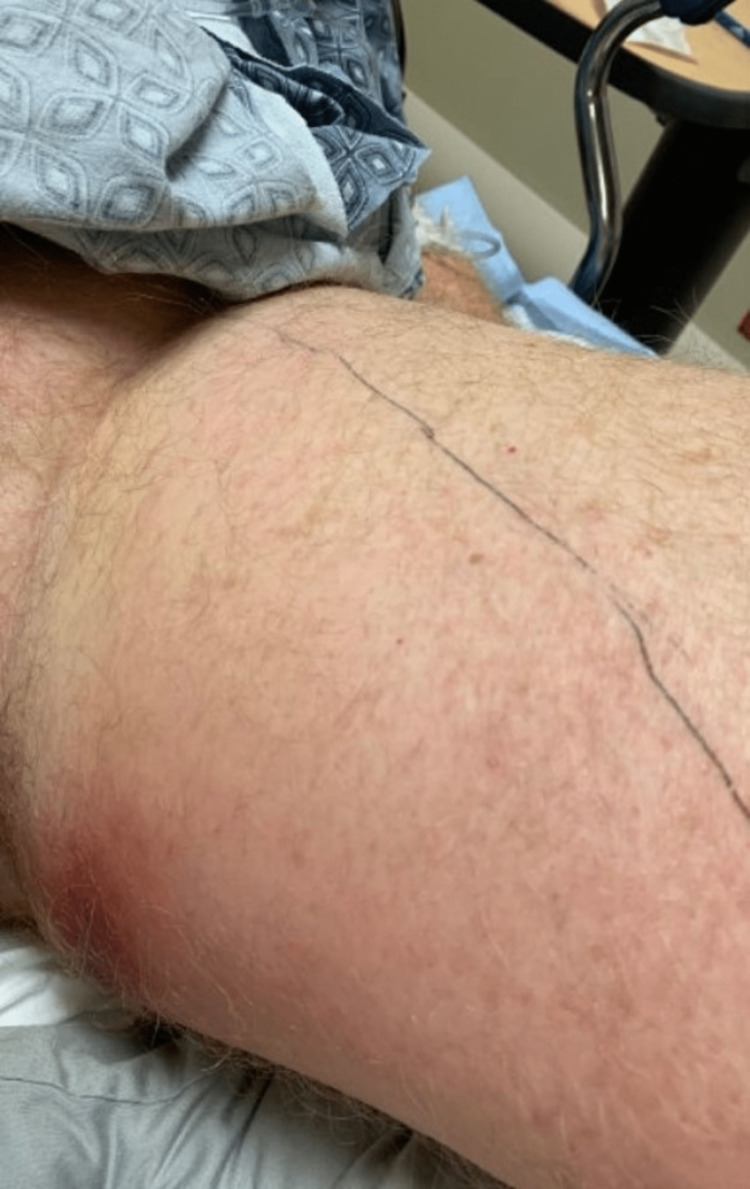
Patient’s left leg on the day he arrived at our institution. Note the extensive edema and erythema on the medial aspect of the proximal thigh.

Physical examination of the affected leg raised concerns for septic arthritis (Figure 2), prompting an orthopedics consultation.

**Figure 3:**
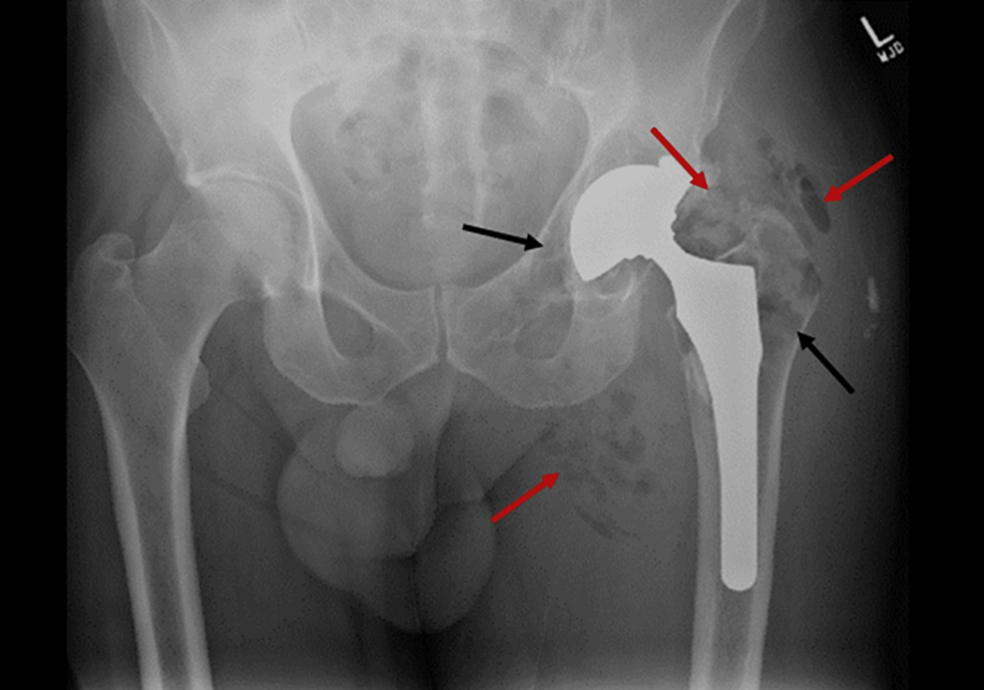
Extensive soft tissue (red arrows) and intraosseous (black arrows) gas around the left hip and hemipelvis, surrounding the left total hip arthroplasty.

Repeat X-ray of the left hip and pelvis demonstrated findings consistent with a periprosthetic necrotizing joint infection (Figure 3). 

Eight days after his initial presentation to the outside emergency department, he was taken to the operating room for irrigation and debridement, where extensive purulence was noted intraoperatively within the joint space and the left thigh medial compartment. Cultures taken during the procedure grew *Salmonella* spp. on two different samples.

Three days after surgical debridement, he underwent implant removal and antibiotic spacer placement. He also required an open reduction and internal fixation of a left femur fracture resulting from significant bone loss with the placement of cerclage cables and screws. All blood cultures at our institution remained negative, and there was no evidence of endovascular involvement. Notably, blood cultures were taken just before his transfer to our institution, which ultimately grew *Salmonella *spp. consistent with his operative cultures.

Despite initially denying a history of fevers or other systemic symptoms in the weeks or months before presenting, the patient later recalled having a gastroenteritis-like illness with watery diarrhea and malaise approximately one week before his current symptoms started.

The patient’s cultures from both our institution and the prior institution were sent to a state laboratory for further analysis and speciation. Our team communicated with the state lab, which reported that whole-genome sequencing was unfortunately not performed given that the patient’s case was not being actively investigated as an outbreak. Therefore, no further species information could be obtained.

Before the initial cultures, the patient was on cefepime 2 g intravenously every eight hours, metronidazole 500 mg intravenously every eight hours, and vancomycin 1500 mg intravenously every 12 hours. Once sensitivities were obtained (Table 2), antibiotics were narrowed to ceftriaxone 2g every 24 hours, with plans for six weeks of therapy followed by oral suppressive therapy with amoxicillin 875 mg orally twice daily based on sensitivity results and to avoid potential drug-drug interactions.

**Table 2:** Susceptibilities of the cultured Salmonella spp. MIC: Minimum inhibitory concentration

Drug	MIC	Susceptibility
Ampicillin	<=2	Susceptible
Ceftazidime	<=1	Susceptible
Ceftriaxone	<=1	Susceptible
Trimethoprim/Sulfamethoxazole	<=20	Susceptible
Ciprofloxacin E Test	0.02	Susceptible
Levofloxacin E Test	0.01	Susceptible

**Figure 4:**
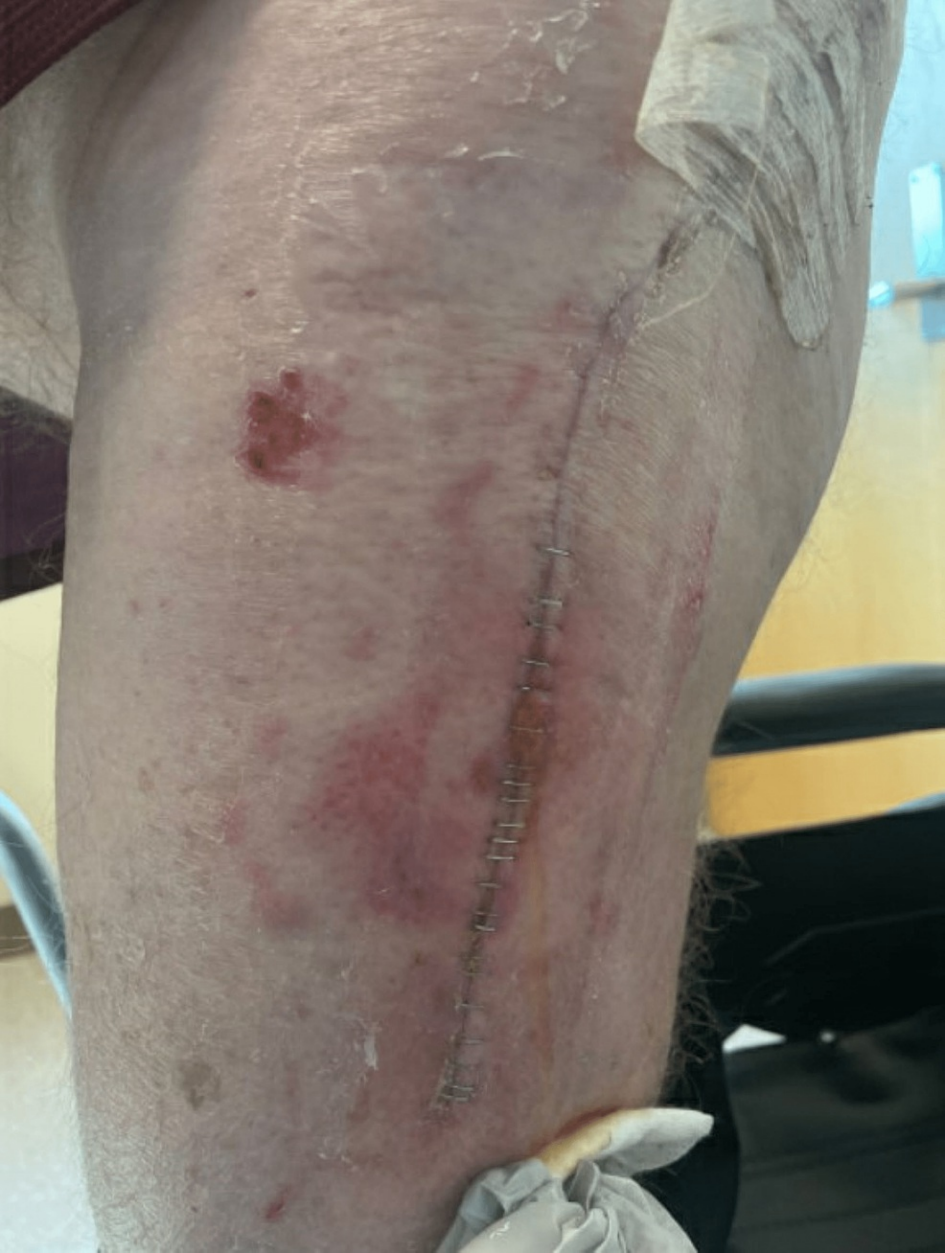
Patient’s left leg approximately one month after irrigation and drainage.

Approximately three weeks after surgery, the patient developed a seroma and required repeat surgery for drainage. There were no signs of infection intra-operatively. He did well clinically, and a repeat examination of the affected leg one month after his initial surgery showed no signs of active infection (Figure 4).

After completing his ceftriaxone regimen, he was transitioned to amoxicillin 875 mg orally twice a day for suppressive therapy. Seven months after his initial surgery, a repeat left hip joint aspiration had no growth on culture, and alpha-defensins testing was negative. He subsequently underwent a left revision total hip arthroplasty and is doing well post-operatively.

## Discussion

*Salmonella *serovars are a common cause of foodborne illness worldwide and are responsible for an estimated 93.8 million cases and 155,000 deaths annually worldwide [1]. After ingestion, *Salmonella *spp. compete with the gut microbiota and colonize the apical epithelium of the intestine, inducing inflammatory changes [2]. Adherence to the intestinal epithelium is assisted by fimbriae and biofilm production [8]. *Salmonella *infection typically presents as gastroenteritis with symptoms, including nausea, vomiting, diarrhea, fever, and abdominal cramping [9]. Given its ability to survive intracellularly within phagocytes, *Salmonella *can also replicate within the reticuloendothelial system and disseminate more widely [10]. While occurring less commonly, bacteria can also spread to multiple distant locations in the body. Sickle-cell disease patients, for example, can develop *Salmonella *osteomyelitis [11,12]. There are also reports of *Salmonella *spp. causing epididymo-orchitis and resulting in abscesses in the parotid gland, ovaries, breast, thyroid, and brain [11-16]. While these infections occur largely in immunocompromised individuals, they have also been observed in otherwise healthy individuals [3,4].

Salmonellosis is a zoonotic disease associated with the consumption of animal products, although other sources, such as vegetables and dietary supplements, have also been implicated in transmission [17,18]. This organism has many animal reservoirs in the agricultural industry, and the development of antibiotic resistance remains a significant threat [5]. For example, a European Union Summary Report on *Salmonella *spp. associated with patient infections, animals, and food production found high rates of resistance to commonly used antimicrobials. According to the European Union Summary report, rates of resistance to ampicillin, sulfonamides, and tetracyclines in the European Union in 2020 ranged from 30%-31% in human cases and 19%-53% in food-producing animals. Resistance to fluoroquinolones in 19%-69% of poultry and turkey samples from 2020 was also noted, along with increasing resistance in human cases between 2016-2020. In addition, multidrug-resistant strains were found in 25% of human cases and 19%-54% of animals used for food production [19]. Studies such as this highlight the growing risk of drug-resistant *Salmonella *spp. pose, especially in immunocompromised patients. In this case, the *Salmonella* strain that infected our patient was fortunately sensitive to the antibiotics given, and the patient had an excellent therapeutic outcome.

## Conclusions

*Salmonella *spp. can cause significant morbidity and mortality, especially in immunocompromised hosts. Increasing antibiotic resistance in serovars pathogenic to humans and animals is of significant concern and may impact the treatment of salmonellosis in the future. Our case illustrates the varied presentation of disseminated infection and serves as a reminder that physicians should consider *Salmonella *infection in their differential diagnosis, particularly in patients with a history of recent gastrointestinal illness.
